# Supplementary material for: HIVprotI: an integrated web based platform for prediction and design of HIV proteins inhibitors
Source: J Cheminform. 2018 Mar 9;10:12. doi: 10.1186/s13321-018-0266-y (PMC5845081; doi:10.1186/s13321-018-0266-y)
Supplement: Supplementary file 2 — Additional file 2. Source code of HIVProtI web server. [file 13321_2018_266_MOESM2_ESM.zip › HIVprotI_Source-code/hivprotI/weka-3-6-6/doc/weka/classifiers/functions/supportVector/CheckKernel.html]

CheckKernel


---


|  |  |  |
| --- | --- | --- |
| **Overview** \| **Package** \| **Class** \| **Tree** \| **Deprecated** \| **Index** \| **Help** \| **Weka's home** | |  |
| **PREV CLASS**   **NEXT CLASS** | **FRAMES**    **NO FRAMES**     **All Classes** |  |
| SUMMARY: NESTED \| FIELD \| CONSTR \| METHOD | DETAIL: FIELD \| CONSTR \| METHOD |  |


---


## weka.classifiers.functions.supportVector Class CheckKernel

```
java.lang.Object
  weka.core.Check
      weka.core.CheckScheme
          weka.classifiers.functions.supportVector.CheckKernel
```

**All Implemented Interfaces:**: OptionHandler, RevisionHandler

---

``` public class CheckKernel extends CheckScheme ```

Class for examining the capabilities and finding problems with
kernels. If you implement an kernels using the WEKA.libraries,
you should run the checks on it to ensure robustness and correct
operation. Passing all the tests of this object does not mean
bugs in the kernels don't exist, but this will help find some
common ones.

Typical usage:

`java weka.classifiers.functions.supportVector.CheckKernel -W kernel_name
-- kernel_options` 

CheckKernel reports on the following:

- Kernel abilities
  - Possible command line options to the kernels
  - Whether the kernels can predict nominal, numeric, string,
    date or relational class attributes.
  - Whether the kernels can handle numeric predictor attributes
  - Whether the kernels can handle nominal predictor attributes
  - Whether the kernels can handle string predictor attributes
  - Whether the kernels can handle date predictor attributes
  - Whether the kernels can handle relational predictor attributes
  - Whether the kernels can handle multi-instance data
  - Whether the kernels can handle missing predictor values
  - Whether the kernels can handle missing class values
  - Whether a nominal kernels only handles 2 class problems
  - Whether the kernels can handle instance weights
- Correct functioning
  - Correct initialisation during buildKernel (i.e. no result
    changes when buildKernel called repeatedly)
  - Whether the kernels alters the data passed to it
    (number of instances, instance order, instance weights, etc)
- Degenerate cases
  - building kernels with zero training instances
  - all but one predictor attribute values missing
  - all predictor attribute values missing
  - all but one class values missing
  - all class values missing

Running CheckKernel with the debug option set will output the
training and test datasets for any failed tests.

The `weka.classifiers.AbstractKernelTest` uses this
class to test all the kernels. Any changes here, have to be
checked in that abstract test class, too.


Valid options are:

```
 -D
  Turn on debugging output.
```

```
 -S
  Silent mode - prints nothing to stdout.
```

```
 -N <num>
  The number of instances in the datasets (default 20).
```

```
 -nominal <num>
  The number of nominal attributes (default 2).
```

```
 -nominal-values <num>
  The number of values for nominal attributes (default 1).
```

```
 -numeric <num>
  The number of numeric attributes (default 1).
```

```
 -string <num>
  The number of string attributes (default 1).
```

```
 -date <num>
  The number of date attributes (default 1).
```

```
 -relational <num>
  The number of relational attributes (default 1).
```

```
 -num-instances-relational <num>
  The number of instances in relational/bag attributes (default 10).
```

```
 -words <comma-separated-list>
  The words to use in string attributes.
```

```
 -word-separators <chars>
  The word separators to use in string attributes.
```

```
 -W
  Full name of the kernel analysed.
  eg: weka.classifiers.functions.supportVector.RBFKernel
  (default weka.classifiers.functions.supportVector.RBFKernel)
```

```
 Options specific to kernel weka.classifiers.functions.supportVector.RBFKernel:
```

```
 -D
  Enables debugging output (if available) to be printed.
  (default: off)
```

```
 -no-checks
  Turns off all checks - use with caution!
  (default: checks on)
```

```
 -C <num>
  The size of the cache (a prime number), 0 for full cache and 
  -1 to turn it off.
  (default: 250007)
```

```
 -G <num>
  The Gamma parameter.
  (default: 0.01)
```

Options after -- are passed to the designated kernel.

**Version:**
:   $Revision: 1.3 $

**Author:**
:   Len Trigg (trigg@cs.waikato.ac.nz), FracPete (fracpete at waikato dot ac dot nz)

**See Also:**: `TestInstances`

---

**Nested Class Summary**

**Nested classes/interfaces inherited from class weka.core.CheckScheme**

`CheckScheme.PostProcessor`


**Constructor Summary**

`CheckKernel()`


| **Method Summary** | |
| --- | --- |
| `void` | `doTests()`             Begin the tests, reporting results to System.out |
| `Kernel` | `getKernel()`             Get the kernel being tested |
| `java.lang.String[]` | `getOptions()`             Gets the current settings of the CheckKernel. |
| `java.lang.String` | `getRevision()`             Returns the revision string. |
| `java.util.Enumeration` | `listOptions()`             Returns an enumeration describing the available options. |
| `static void` | `main(java.lang.String[] args)`             Test method for this class |
| `void` | `setKernel(Kernel value)`             Set the lernel to test. |
| `void` | `setOptions(java.lang.String[] options)`             Parses a given list of options. |

**Methods inherited from class weka.core.CheckScheme**

`attributeTypeToString, getNumDate, getNumInstances, getNumInstancesRelational, getNumNominal, getNumNumeric, getNumRelational, getNumString, getPostProcessor, getWords, getWordSeparators, hasClasspathProblems, setNumDate, setNumInstances, setNumInstancesRelational, setNumNominal, setNumNumeric, setNumRelational, setNumString, setPostProcessor, setWords, setWordSeparators`

**Methods inherited from class weka.core.Check**

`getDebug, getSilent, setDebug, setSilent`

**Methods inherited from class java.lang.Object**

`equals, getClass, hashCode, notify, notifyAll, toString, wait, wait, wait`

**Constructor Detail**

### CheckKernel

```
public CheckKernel()
```


**Method Detail**

### listOptions

```
public java.util.Enumeration listOptions()
```

:   Returns an enumeration describing the available options.

    :   **Specified by:**: `listOptions` in interface `OptionHandler` **Overrides:**: `listOptions` in class `CheckScheme`
    :   **Returns:**: an enumeration of all the available options.

---


### setOptions

```
public void setOptions(java.lang.String[] options)
                throws java.lang.Exception
```

:   Parses a given list of options.
    Valid options are:

    ```
     -D
      Turn on debugging output.
    ```

    ```
     -S
      Silent mode - prints nothing to stdout.
    ```

    ```
     -N <num>
      The number of instances in the datasets (default 20).
    ```

    ```
     -nominal <num>
      The number of nominal attributes (default 2).
    ```

    ```
     -nominal-values <num>
      The number of values for nominal attributes (default 1).
    ```

    ```
     -numeric <num>
      The number of numeric attributes (default 1).
    ```

    ```
     -string <num>
      The number of string attributes (default 1).
    ```

    ```
     -date <num>
      The number of date attributes (default 1).
    ```

    ```
     -relational <num>
      The number of relational attributes (default 1).
    ```

    ```
     -num-instances-relational <num>
      The number of instances in relational/bag attributes (default 10).
    ```

    ```
     -words <comma-separated-list>
      The words to use in string attributes.
    ```

    ```
     -word-separators <chars>
      The word separators to use in string attributes.
    ```

    ```
     -W
      Full name of the kernel analysed.
      eg: weka.classifiers.functions.supportVector.RBFKernel
      (default weka.classifiers.functions.supportVector.RBFKernel)
    ```

    ```
     Options specific to kernel weka.classifiers.functions.supportVector.RBFKernel:
    ```

    ```
     -D
      Enables debugging output (if available) to be printed.
      (default: off)
    ```

    ```
     -no-checks
      Turns off all checks - use with caution!
      (default: checks on)
    ```

    ```
     -C <num>
      The size of the cache (a prime number), 0 for full cache and 
      -1 to turn it off.
      (default: 250007)
    ```

    ```
     -G <num>
      The Gamma parameter.
      (default: 0.01)
    ```

    :   **Specified by:**: `setOptions` in interface `OptionHandler` **Overrides:**: `setOptions` in class `CheckScheme`
    :   **Parameters:**: `options` - the list of options as an array of strings **Throws:**: `java.lang.Exception` - if an option is not supported

---


### getOptions

```
public java.lang.String[] getOptions()
```

:   Gets the current settings of the CheckKernel.

    :   **Specified by:**: `getOptions` in interface `OptionHandler` **Overrides:**: `getOptions` in class `CheckScheme`
    :   **Returns:**: an array of strings suitable for passing to setOptions

---


### doTests

```
public void doTests()
```

:   Begin the tests, reporting results to System.out

    :   **Specified by:**: `doTests` in class `CheckScheme`

---


### setKernel

```
public void setKernel(Kernel value)
```

:   Set the lernel to test.

    :   **Parameters:**: `value` - the kernel to use.

---


### getKernel

```
public Kernel getKernel()
```

:   Get the kernel being tested

    :   **Returns:**: the kernel being tested

---


### getRevision

```
public java.lang.String getRevision()
```

:   Returns the revision string.

    :   **Returns:**: the revision

---


### main

```
public static void main(java.lang.String[] args)
```

:   Test method for this class

    :   **Parameters:**: `args` - the commandline parameters


---


|  |  |  |
| --- | --- | --- |
| **Overview** \| **Package** \| **Class** \| **Tree** \| **Deprecated** \| **Index** \| **Help** \| **Weka's home** | |  |
| **PREV CLASS**   **NEXT CLASS** | **FRAMES**    **NO FRAMES**     **All Classes** |  |
| SUMMARY: NESTED \| FIELD \| CONSTR \| METHOD | DETAIL: FIELD \| CONSTR \| METHOD |  |


---
